# A Novel Sparse Framework for Angle and Frequency Estimation

**DOI:** 10.3390/s22228633

**Published:** 2022-11-09

**Authors:** Guilian Zhao, Dongmei Huang, Changxin Cai, Peng Wu

**Affiliations:** 1 School of Electronic and Information, Yangtze University, Jingzhou 434023, China; 2 Department of Training Management, Naval Command College, Nanjing 210016, China

**Keywords:** angle estimation, frequency estimation, coprime sampling, rotational invariance, closed-form solution

## Abstract

The topic of joint angle and frequency estimation (JAFE) has aroused extensive interests in the past decades. Current estimation algorithms mainly rely on the Nyquist sampling criterion. In order not to cause ambiguity for parameter estimation, the space–time intervals must be smaller than given thresholds, which results in complicated hardware costs and a huge computational burden. This paper aims to reduce the complexity for JAFE, and a novel sparsity-aware framework is proposed. Unlike the current uniform sampling architectures, the incoming narrow-band singles are sampled by a series of space–time coprime samplers. An improved rotational invariance estimator is introduced, which offers closed-form solutions for both angle and frequency estimation. The mathematical treatments indicate that our methodology is inherent in larger spatial/temporal aperture than the uniform sampling architectures; hence, it provides more accurate JAFE compared to alternative approaches relying on uniform sampling. Additionally, it attains nearly the same complexity as the current rotational invariance approach. Numerical results agree with the theoretical advantages of our methodology.

## 1. Introduction

In cognitive wireless systems [1,2], a critical ingredient is to obtain the super-resolution angle and frequency estimation of the incoming sources [3]. With the priors of the estimated angle and frequency, many smart technologies are applicable, e.g., beamforming [4,5] and intelligence spectrum allocation [6]. Many estimation strategies have been proposed [7], for instance, multiple signal classification (MUSIC), the estimation method of signal parameters via rotational invariance technique (ESPRIT), tensor-aware approaches, and optimization-based methods. Among the existing algorithms, ESPRIT is appealing since it provides closed-form results for parameter estimation. Comparatively speaking, MUSIC and the tensor approaches are less efficient due to the exhaustive search or complicated matrix decomposition. Additinally, the deep learning technique is attractive from the perspective of estimation performance and computational complexity [8], but a large amount of time is required for off-line learning.

The well-known Nyquist–Shannon sampling theorem has guided signal sampling for more than sixty years. The Nyquist–Shannon sampling theorem is also called the uniform sampling theorem. It pointed out that with the highest frequency *f* that can be undistorted and recovered from its samplers, it can be uniformly sampled with an interval smaller than 1/(2f). Numerous investigations on JAFE are based on the uniform space–time sampling architecture, which consists of an *M*-element uniform linear array (ULA) and equal number of time delay groups. In order not to cause ambiguity, the sampling in both the spatial domain and temporal domain must obey the Nyquist–Shannon theorem. In other words, the inter-element distance must be no more than half the wavelength, and the time interval of two adjacent delayers must be no more than half of the smallest source period. Various strategies have been introduced for the estimation issue with such uniform sampling. In [9], an ESPRIT algorithm was reported, which utilizes the uniformity of the space–time sampling and offers closed-form solutions for JAFE. In order to exploit the multi-dimensional nature of the space–time measurement, a parallel factor (PARAFAC) algorithm was investigated in [10]. Considering the symmetrical characteristic of the space–time sampling, a real-valued PARAFAC estimator was introduced in [11], which is suitable for coherent sources and has less computational burden than the one documented in [10].

To pursue super-resolution JAFE, one effective solution is to increase the space–time sampling terminals. However, massive sampling terminals would bring an extreme hardware burden to the traditional Nyquist sampling-based framework. To reduce the hardware cost, the spatial compressive sampling strategy has been frequently discussed. In [12,13], the random compressive matrix idea was carried out to reduce the required number of front-end circuits. However, the random sampler would cause a significant performance loss. An alternative to the random sampling architecture is the sparse uniform framework, which has a lower hardware cost while providing comparable performance compared to the Nyquist sampling method. Typical sparse uniform sampling approaches include nested sampling and coprime sampling [14,15]. Additionally, a sparse uniform linear array with a much larger distance and auxiliary information is also attractive [16]. The sparse sampling has became a hot topic in angle estimation, and the sparse sampling idea brings new insight to JAFE. Unlike the traditional uniform sampling architecture, a sparse framework can greatly reduce the spatial/temporal measurement. The temporal compressive methods were discussed in [17,18], which can greatly reduce the data rate in the temporal domain. Nevertheless, the sparse recovery mechanisms are computationally inefficient. A multi-way compressive sampling framework was discussed in [19], which obtains the low-dimensional space–time measurement via two random compressive matrices. Although it has less computational complexity than [10], it suffers from performance loss. In [20], a nested sampling scheme was introduced, in which the sources were discretized using nested samplers in both the spatial domain and temporal domain, namely the sensor array geometry satisfies the nested structure, and the time interval of the delayers obeys the nested principle. Therein, the two-dimensional pseudospectrum search approach was proposed for JAFE from the sub-Nyquist measurement. Unfortunately, the spatial nested array may suffer from unknown mutual coupling, and the grid search strategy is barely realizable for real-time implementation.

A coprime array consists of two ULAs, which have *M* and *N* elements. Herein, *M* and *N* are coprime numbers, and the distances of the two ULAs are Nd and Md, respectively. Although there is ambiguity in the angle estimation for each ULA, this ambiguity can be eliminated via the coprime characteristic. It is well-known that a coprime geometry would sustain less mutual coupling than a nested manifold in the spatial domain [15,21], and the former requires a smaller data volume than the latter in the temporal domain. To the best of our knowledge, a coprime array was proposed in [3] for JAFE, but the sparse sampling took place only in the spatial domain. Inspired by the advantages of coprime sampling, a novel space–time coprime sampling framework is proposed for JAFE. More exactly, the main contributions of this paper are highlighted as follows:

- A novel spatial-time sparse sampling architecture is introduced. The proposed framework consists of a coprime linear array and coprime-distributed delayers. Unlike the existing spatial sparse sampling architecture that only samples the signal in the spatial domain, the sparse sampling is not only carried out in the spatial domain, but is also carried out in the time domain. Namely, much less measurements are required in the proposed framework.
- An ESPRIT-like estimator is developed for JAFE from the spatial-time spare measurement. The proposed algorithm first obtains the ambiguous estimates via the uniformity of the space–time subarrays. Thereafter, it determines the unique JAFE by exploiting the coprime characteristic of the subarrays. It can offer a closed-form solution for JAFE, and it defeats the current uniform sampling architectures in terms of estimation accuracy.
- The proposed estimator is analyzed in terms of spatial/temporal aperture, computational complexity, and the theoretical lower bound and numerical simulations corroborate the theoretical advantages.

For convenience, the definitions of the following symbols are given. $A^{T}$, $A^{H}$, $A^{†}$ and $A^{-1}$ denote, respectively, the transpose, Hermitian transpose, pseudo-inverse and inverse of A; ⊗, ⊙ and ⊕ denote Kronecker product, KhatriRao product and Hadamard product, respectively; $I_{M}$ denotes the M×M identity matrix, while $0_{M\times N}$ and $1_{M}$ denote an M×N full zeros matrix and an M×M full ones matrix, respectively; $\mathrm{diag}\left\{\cdot \right\}$, $E\left\{\cdot \right\}$, $\mathrm{phase}\left\{\cdot \right\}$ and $\mathrm{real}\left(\cdot \right)$ represent diagonalization, expectation, phase and real part, respectively.

## 2. Problem Formulation

**Figure 1:**
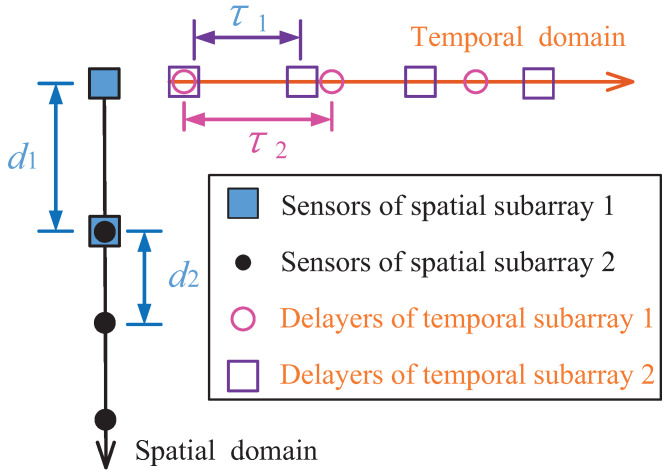
An example of the proposed space–time coprime sampling framework, where $M_{1}=2$, $M_{2}=3$, $N_{1}=4$, $N_{2}=3$.

The proposed space–time coprime sampling framework is illustrated in Figure 1. The sampling hardware consist of an *M*-element ($M=M_{1}+M_{2}-1$, $M_{1}$ and $M_{2}$ are coprime numbers) coprime sensor array and *M* time delay groups. The coprime sensor array is composed of an $M_{1}$-element ULA and an $M_{2}$-element ULA (the two ULAs share the same reference sensor), the inter-element distances of which are, respectively, $d_{1}=M_{2}d$ and $d_{2}=M_{1}d$, where *d* denotes a standard inter-element distance of the traditional ULA (*d* should be chosen so that it is smaller than half of the smallest wavelength of the sources). For *K* narrow band, far-field sources impacting on the sensors, the response of the sensor array is represented as:

(1) $$x_{0}\left(t\right)=\mathrm{As}\left(t\right)+n_{0}\left(t\right)$$

where *t* denotes the time index, $A=\left[a_{1},a_{2},\cdots ,a_{k}\right]\in C^{M\times K}$ denotes the spatial response matrix with $a_{k}=\left[u_{k}^{-\left(M_{1}-1\right)M_{2}},u_{k}^{-\left(M_{1}-2\right)M_{2}},\cdots ,1,u_{k}^{M_{1}}\right.$, ${\left.\cdots ,u_{k}^{\left(M_{2}-1\right)M_{1}}\right]}^{T}$, $u_{k}=e^{-j2\pi df_{k}\sin\theta_{k}}$, $f_{k}$ and $\theta_{k}$ denote the carrier frequency and direction-of-arrival (DOA) of the *k*-th source; $s\left(t\right)\in C^{K\times 1}$ stands for the source vector; $n_{0}\left(t\right)\in C^{M\times 1}$ denotes the additive white Gaussian noise vector with power $\sigma^{2}$. After delay p▵ (▵ denotes a standard interval of the traditional uniform delay network and $▵<1/2\max\left\{f_{k}\right\}$, *p* is a integer), (1) can be formulated as:

(2) $$\begin{array}{ccc} x_{p}\left(t\right) & = & x\left(t-p▵\right)\\ & = & \mathrm{As}\left(t-p▵\right)+n_{0}\left(t-p▵\right)\\ & = & {\mathrm{AV}}^{p}s\left(t\right)+n_{p}\left(t\right) \end{array}$$

where $V=\mathrm{diag}\left\{v_{1},v_{2},\cdots ,v_{K}\right\}$, $v_{k}=e^{-j2\pi f_{k}▵}$. To estimate the carrier frequency, *M* time delay groups are connected with the sensor array. Each time delay group is made up of two uniform delay arrays, which have, respectively, $N_{1}$ and $N_{2}$ delayers ($N_{1}$ and $N_{2}$ are also coprime numbers), and the corresponding delay intervals are $\tau_{1}=N_{2}▵$ and $\tau_{2}=N_{1}▵$. By arranging

(3) $$X\left(t\right)=\left[\begin{array}{c} x_{\left(N_{1}-1\right)N_{2}}\left(t\right)\\ x_{\left(N_{1}-2\right)N_{2}}\left(t\right)\\ \;\;\;\vdots \\ \;\;x_{0}\left(t\right)\\ \;x_{N_{1}}\left(t\right)\\ \;\;\;\vdots \\ x_{\left(N_{2}-1\right)N_{1}}\left(t\right) \end{array}\right],N\left(t\right)=\left[\begin{array}{c} n_{\left(N_{1}-1\right)N_{2}}\left(t\right)\\ n_{\left(N_{1}-2\right)N_{2}}\left(t\right)\\ \;\;\;\vdots \\ \;\;n_{0}\left(t\right)\\ \;n_{N_{1}}\left(t\right)\\ \;\;\;\vdots \\ n_{\left(N_{2}-1\right)N_{1}}\left(t\right) \end{array}\right]$$

we have:

(4) $$\begin{array}{ccc} X\left(t\right) & = & \left[A⊙B\right]s\left(t\right)+N\left(t\right)\\ & = & \mathrm{Cs}\left(t\right)+N\left(t\right) \end{array}$$

where $B=\left[b_{1},b_{2},\cdots ,b_{k}\right]\in C^{N\times K}$ denotes the temporal response matrix with $b_{k}={\left[v_{k}^{\left(N_{1}-1\right)N_{2}},v_{k}^{\left(N_{1}-2\right)N_{2}},\cdots ,1,v_{k}^{N_{1}},\cdots ,v_{k}^{\left(N_{2}-1\right)N_{1}}\right]}^{T}$, C=A⊙B.

When the sources are uncorrelated, the covariance matrix of $X\left(t\right)$ will be represented as:

(5) $$\begin{array}{ccc} R & = & E\{X\left(t\right)X^{H}\left(t\right)\}\\ & = & CR_{s}C^{H}+\sigma^{2}I_{MN} \end{array}$$

where $R_{s}=E\left\{s\left(t\right)s^{H}\left(t\right)\right\}$ represents the sources’ covariance matrix. Usually, R can be estimated via *L* measurements as:

(6) $$\begin{array}{c} \hat{R}=1/L\sum_{t=1}^{L}X\left(t\right)X^{H}\left(t\right) \end{array}$$

From the eigenvalue decomposition of R one can obtain the signal subspace $E_{s}$ (which consists of the eigenvectors with respect to the *K* dominate eigenvalues). It is well-known that $E_{s}$ spans the same space with C, namely:

(7) $$E_{s}=CT$$

where $T\in C^{K\times K}$ represents a full rank matrix.

## 3. Joint DOA and Frequency Estimation

### 3.1. Ambiguous DOA and Frequency Estimation

Define $J_{d,1}=\left[0_{(M_{1}-1)\times 1},I_{\left(M_{1}-1\right)},0_{\left(M_{1}-1\right)\times \left(M_{2}-1\right)}\right]\in C^{(M_{1}-1)\times M}$, $J_{d,2}=\left[I_{\left(M_{1}-1\right)}\right.$, $\left.0_{\left(M_{1}-1\right)\times M_{2}}\right]\in C^{(M_{1}-1)\times M}$, $J_{d,3}≜[0_{\left(M_{2}-1\right)\times \left(M_{1}-1\right)}$, $I_{\left(M_{2}-1\right)},0_{(M_{2}-1)\times 1}]\in C^{(M_{2}-1)\times M}$, $J_{d,4}=\left[0_{\left(M_{2}-1\right)\times M_{1}},I_{\left(M_{2}-1\right)}\right]\in C^{(M_{2}-1)\times M}$, $J_{f,1}=\left[0_{(N_{1}-1)\times 1},I_{\left(N_{1}-1\right)},0_{\left(N_{1}-1\right)\times \left(N_{2}-1\right)}\right]\in C^{(N_{1}-1)\times N}$, $J_{f,2}=\left[I_{\left(N_{1}-1\right)},0_{\left(N_{1}-1\right)\times N_{2}}\right]\in C^{(N_{1}-1)\times N}$, $J_{f,3}=\left[0_{\left(N_{2}-1\right)\times \left(N_{1}-1\right)},I_{\left(N_{2}-1\right)}\right.$, $\left.0_{(N_{2}-1)\times 1}\right]\in C^{(N_{2}-1)\times N}$, $J_{f,4}=\left[0_{\left(N_{2}-1\right)\times N_{1}},I_{\left(N_{2}-1\right)}\right]\in C^{(N_{2}-1)\times N}$. From the rotational invariances of the spatial subarrays as well as the temporal subarrays, we have:

(8a) $$J_{d,1}A=J_{d,2}A\Psi_{1}$$

(8b) $$J_{d,3}A=J_{d,4}A\Psi_{2}$$

(8c) $$J_{f,1}B=J_{f,2}B\Psi_{3}$$

(8d) $$J_{f,3}B=J_{f,4}B\Psi_{4}$$

where $\Psi_{1}=\mathrm{diag}\left\{u_{1}^{M_{2}},u_{2}^{M_{2}}\cdots ,u_{K}^{M_{2}}\right\}$, $\Psi_{2}=\mathrm{diag}\left\{u_{1}^{M_{1}},u_{2}^{M_{1}}\cdots ,u_{K}^{M_{1}}\right\}$, $\Psi_{3}=\mathrm{diag}\left\{v_{1}^{N_{2}}\right.$, $\left.v_{2}^{N_{2}}\cdots ,v_{K}^{N_{2}}\right\}$, $\Psi_{4}=\mathrm{diag}\left\{v_{1}^{N_{1}},v_{2}^{N_{1}}\cdots ,v_{K}^{N_{1}}\right\}$. Therefore, we define $H_{d,m}=J_{d,1}⊗I_{N}$, $H_{f,m}=I_{M}⊗J_{f,m}$, m=1,2,3,4. It is easy to obtain:

(9a) $$H_{d,1}C=H_{d,2}C\Psi_{1}$$

(9b) $$H_{d,3}C=H_{d,4}C\Psi_{2}$$

(9c) $$H_{f,1}C=H_{f,2}C\Psi_{3}$$

(9d) $$H_{f,3}C=H_{f,4}C\Psi_{4}$$

Let $E_{d,m}=H_{d,m}E_{s}$, $E_{f,m}=H_{f,m}E_{s}$. Replacing C with $E_{s}T^{-1}$ yields:

(10a) $$E_{d,2}^{†}E_{d,1}=T^{-1}\Psi_{1}T$$

(10b) $$TE_{d,4}^{†}E_{d,3}T^{-1}=\Psi_{2}$$

(10c) $$TE_{f,2}^{†}E_{f,1}T^{-1}=\Psi_{3}$$

(10d) $$TE_{f,4}^{†}E_{f,3}T^{-1}=\Psi_{4}$$

From (10a) one can conclude that the diagonal entities of $\Psi_{1}$ are the eigenvalues of $E_{d,2}^{†}E_{d,1}$, and the *k*-th column vector of T is an eigenvector corresponding to the *k*-th eigenvalue of $E_{d,2}^{†}E_{d,1}$. Therefore, the estimates of $\Psi_{1}$ and T, denoted as ${\hat{\Psi }}_{1}$ and $\hat{T}$, respectively, can be obtained via the eigendecomposition of $E_{d,2}^{†}E_{d,1}$. Thereafter, replace T with $\hat{T}$, and calculate the left sides of (10); then, we can achieve the estimates of $\Psi_{2}$, $\Psi_{3}$ and $\Psi_{4}$, which are denoted by ${\hat{\Psi }}_{2}$, ${\hat{\Psi }}_{3}$ and ${\hat{\Psi }}_{4}$, respectively. Since $\Psi_{1}$, $\Psi_{2}$, $\Psi_{3}$ and $\Psi_{4}$ share the same perturbed matrix T, their estimates ${\hat{\Psi }}_{1}$, ${\hat{\Psi }}_{2}$, ${\hat{\Psi }}_{3}$ and ${\hat{\Psi }}_{4}$ are paired automatically. Denote the *k*-th diagonals of ${\hat{\Psi }}_{1}$, ${\hat{\Psi }}_{2}$, ${\hat{\Psi }}_{3}$ and ${\hat{\Psi }}_{4}$ as $\alpha_{1}$, $\alpha_{2}$, $\alpha_{3}$ and $\alpha_{4}$, respectively. Then, $f_{k}$ and $\theta_{k}$ can be achieved via:

(11a) $${\hat{f}}_{k,1}=-\frac{\mathrm{angle}\left(\alpha_{1}\right)}{2\pi N_{2}▵}$$

(11b) $${\hat{f}}_{k,2}=-\frac{\mathrm{angle}\left(\alpha_{2}\right)}{2\pi N_{1}▵}$$

(11c) $${\hat{\theta }}_{k,1}=-\frac{\mathrm{angle}\left(\alpha_{3}\right)}{2\pi M_{2}d{\hat{f}}_{k}}$$

(11d) $${\hat{\theta }}_{k,2}=-\frac{\mathrm{angle}\left(\alpha_{4}\right)}{2\pi M_{1}d{\hat{f}}_{k}}$$

However, the ranges of $2\pi N_{2}f_{k}▵$, $2\pi N_{1}f_{k}▵$, $2\pi M_{2}d{\hat{f}}_{k}\sin\theta_{k}$ and $2\pi M_{1}d{\hat{f}}_{k}\sin\theta_{k}$ are within $\left[-N_{2}\pi ,N_{2}\pi \right]$, $\left[-N_{1}\pi ,N_{1}\pi \right]$, $\left[-M_{2}\pi ,M_{2}\pi \right]$ and $\left[-M_{1}\pi ,M_{1}\pi \right]$, respectively, but $\mathrm{angle}\left(\cdot \right)$ is within the interval $\left[-\pi ,\pi \right]$. Therefore, the estimates in (11) are ambiguous. Since $\Psi_{1}$, $\Psi_{2}$, $\Psi_{3}$ and $\Psi_{4}$ share the same perturbation T, which has been compensated when calculating ${\hat{f}}_{k,1}$, ${\hat{f}}_{k,2}$, ${\hat{\theta }}_{k,1}$ and ${\hat{\theta }}_{k,2}$, all of the estimated parameters are paired automatically.

### 3.2. Unique DOA and Frequency Determination

**Figure 2:**
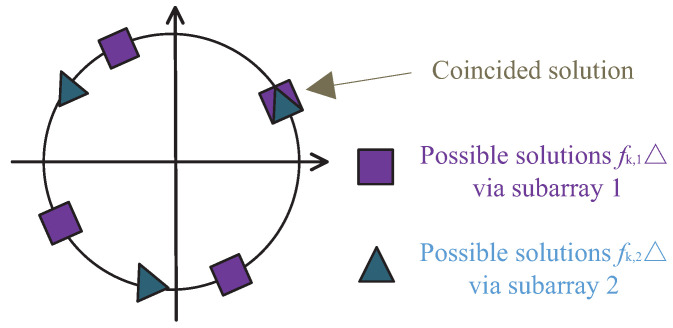
An example of unique frequency determination, where $N_{1}=4$, $N_{2}=3$, $f_{k}▵=0.5$.

The unique JAFE can be accomplished by exploiting the spatial and temporal coprime characteristics. It is well known that $v_{k}=e^{-j2\pi f_{k}p▵}$ is a periodic function of $f_{k}=1/\left(p▵\right)$. Consequently, for the estimated frequencies ${\hat{f}}_{k,1}$ and ${\hat{f}}_{k,2}$ in (11a), there should be totally $N_{2}$ and $N_{1}$ possible solutions (including ${\hat{f}}_{k,1}$, ${\hat{f}}_{k,2}$), respectively, so that:

(12a) $${\hat{f}}_{k,1}^{\left(n2\right)}={\hat{f}}_{k,1}^{\left(1\right)}+\left(n2-1\right)/\left(N_{2}▵\right)\;,\;n2=1,2,\cdots ,N_{2}$$

(12b) $${\hat{f}}_{k,2}^{\left(n1\right)}={\hat{f}}_{k,2}^{\left(1\right)}+\left(n1-1\right)/\left(N_{1}▵\right)\;,\;n1=1,2,\cdots ,N_{1}$$

where ${\hat{f}}_{k,1}^{\left(1\right)}$ and ${\hat{f}}_{k,2}^{\left(1\right)}$ denote the minimum nonnegative values. An example is presented in Figure 2. Finally, the unambiguous frequency estimate ${\hat{f}}_{k}$ can be determined by averaging the nearest ${\hat{f}}_{k,1}^{\left(n2\right)}$ and ${\hat{f}}_{k,2}^{\left(n1\right)}$, i.e.,

(13) $${\hat{f}}_{k}=\left({\hat{f}}_{k,1}^{\left(n2\right)}+{\hat{f}}_{k,2}^{\left(n1\right)}\right)/2\;s.t.\;\min\left|{\hat{f}}_{k,1}^{\left(n2\right)}-{\hat{f}}_{k,2}^{\left(n1\right)}\right|$$

Similarly, there should be, respectively, $M_{2}$ and $M_{1}$ possible candidates (including ${\hat{\theta }}_{k,1}$, ${\hat{\theta }}_{k,2}$) for the estimated DOA in (11b) and fulfill:

(14a) $${\hat{\theta }}_{k,1}^{\left(m2\right)}={\hat{\theta }}_{k,1}^{\left(1\right)}+\left(m2-1\right)/\left(M_{2}d{\hat{f}}_{k}\right)\;,\;m2=1,2,\cdots ,M_{2}$$

(14b) $${\hat{\theta }}_{k,2}^{\left(m1\right)}={\hat{\theta }}_{k,2}^{\left(1\right)}+\left(m1-1\right)/\left(M_{1}d{\hat{f}}_{k}\right)\;,\;m1=1,2,\cdots ,M_{1}$$

Additionally, the unique DOA estimation can be determined via averaging the nearest ${\hat{\theta }}_{k,1}^{\left(m2\right)}$ and ${\hat{\theta }}_{k,1}^{\left(m1\right)}$, i.e.,

(15) $${\hat{\theta }}_{k}=\left({\hat{\theta }}_{k,1}^{\left(n2\right)}+{\hat{\theta }}_{k,2}^{\left(n1\right)}\right)/2\;s.t.\;\min\left|{\hat{\theta }}_{k,1}^{\left(n2\right)}-{\hat{\theta }}_{k,2}^{\left(n1\right)}\right|$$

## 4. Schematic Analysis

### 4.1. Spatial/Temporal Aperture

Since the DOA and the frequency are, respectively, estimated via the rotational invariance of the spatial sensor array and the rotational invariance of the temporal delay network, the spatial/temporal aperture will play an important role in DOA/frequency estimation. For the proposed framework, one can easily conclude that the spatial aperture and the temporal aperture are $\max\left\{M_{1}\left(M_{2}-1\right)d,M_{2}\left(M_{1}-1\right)d\right\}$ and $\max\left\{N_{1}\left(N_{2}-1\right)▵,N_{2}\left(N_{1}-1\right)▵\right\}$, respectively. However, for the traditional uniform sampling schemes, the spatial aperture and the temporal aperture are (M−1)d and (N−1)▵, respectively. In Table 1, we list the aperture comparison between the proposed framework and the current uniform sampling architectures. In actual applications, we have:

(16a) $$\max\left\{M_{1}\left(M_{2}-1\right)d,M_{2}\left(M_{1}-1\right)d\right\}>\left(M-1\right)d$$

(16b) $$\max\left\{N_{1}\left(N_{2}-1\right)▵,N_{2}\left(N_{1}-1\right)▵\right\}>\left(N-1\right)▵$$

Therefore, the proposed framework occupies a larger spatial/temporal aperture than the traditional uniform sampling counterparts. As a result, the proposed framework would provide more accurate estimates than that in [9,10,11].

### 4.2. Complexity Analysis

The dominating complexity of the our framework is the eigendecomposition, which requires $O\left\{M^{3}N^{3}\right\}$ complex multiplications. Additionally, the calculation of ${\hat{\Psi }}_{1}$, ${\hat{\Psi }}_{2}$, ${\hat{\Psi }}_{3}$ and ${\hat{\Psi }}_{4}$ in (10) needs approximate $2{\left(M_{2}-1\right)}^{2}N^{2}K+2{\left(M_{1}-1\right)}^{2}K+\left(M-1\right)NK^{2}+2{\left(N_{2}-1\right)}^{2}M^{2}K+2{\left(N_{1}-1\right)}^{2}M^{2}K+\left(N-1\right)MK^{2}+O\left\{K^{3}\right\}$ complex multiplications. In Table 1, we list the approximate complexities with respect to the ESPRIT approach in [9], the PARAFAC approach in [10] and the unitary PARAFAC (UPARAFAC) estimator in [11]. It is observed that the proposed framework attains very similar complexity to that of ESPRIT, and may be smaller than that of PARAFAC and UPARAFAC in the presence of small *M* and *N* values.

### 4.3. Stability

Stability is an important merit to evaluate an estimator [22]. In this subsection, the stabilities with respect to various algorithms are analyzed. By comparing the algorithmic steps of various algorithms, one can conclude that both ESPRIT and the proposed algorithm are ‘direct’ methods without any iterative steps. However, both PARAFAC and UPARAFAC are carried out in an iterative way. Although the iteration in both PARAFAC and UPARAFAC is easy to be implemented and ensured to converge to the local minimums, there is no theoretical guarantee that proves the local minimums are total minimums. Consequently, both ESPRIT and the proposed algorithm are stable while both PARAFAC and UPARAFAC are not stable.

### 4.4. Cramér–Rao Bound (CRB)

It is easy to obtain the CRB on JAFE via:

(17) $$\mathrm{CRB}=\frac{\sigma^{2}}{2L}{\left[\mathrm{real}\left(\left({\tilde{C}}^{H}\Pi_{C}^{⊥}\tilde{C}\right)⊕\left(R_{s}^{T}⊗1_{2}\right)\right)\right]}^{-1}$$

where $\tilde{C}≜\left[\partial c_{1}/\partial \theta_{1},\partial c_{2}/\partial \theta_{2},\cdots ,\partial c_{K}/\partial \theta_{K},\partial c_{1}/\partial f_{1}\right.$, $\left.\partial c_{2}/\partial f_{2},\cdots ,\partial c_{K}/\partial f_{K}\right]\in C^{MN\times 2K}$, $\Pi_{C}=I_{MN}-CC^{†}$, $c_{k}$ denotes the *k*-th column of C.

## 5. Simulation Results

Computer experiments were implemented to show the improvement of our framework. Herein, we assume that the K=3 far-field sources come from $\theta_{1}=10^{\circ }$, $\theta_{2}=20^{\circ }$ and $\theta_{2}=30^{\circ }$, and the corresponding carrier frequencies are $f_{1}=1$ MHz, $f_{2}=1.5$ MHz and $f_{3}=1.8$ MHz, respectively. The standard inter-element interval *d* is set to $1.25\times 10^{-7}$ m, and the standard delay interval ▵ is fixed at $10^{-7}$ s. Moreover, we assume that the spatial coprime array consists of $M_{1}$ and $M_{2}$ sensors, while the temporal coprime sampler consists of $N_{1}$ and $N_{2}$ delay units, respectively. Each result relies on 500 Monte Carlo trials. The root mean square error (RMSE) is adopted as a accuracy meter (refer to the definition in [11]).

**Figure 3:**
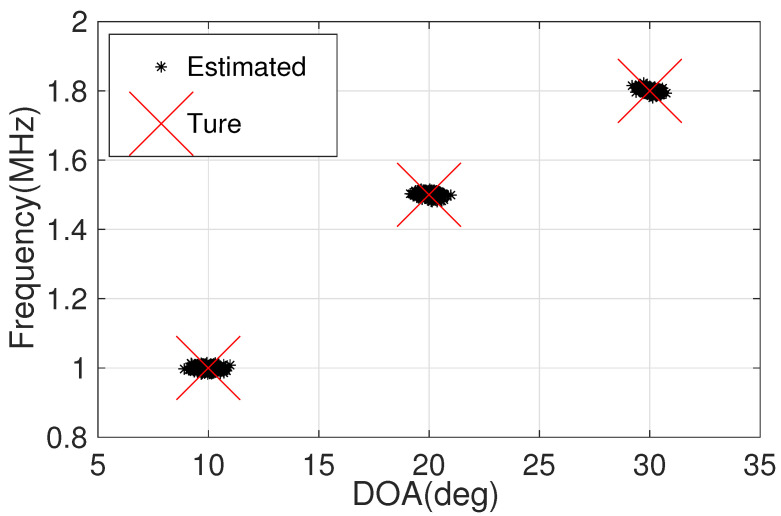
Scatter results of the proposed framework.

Figure 3 describes the scatter result of our framework, where $M_{1}=3$, $M_{2}=4$, $N_{1}=3$, $N_{2}=5$, L=100, and the signal-to-noise ratio (SNR) is fixed at 0 dB. Obviously, both DOAs and frequencies can be accurately recovered and correctly paired. It is effectively shown that our framework is capable of providing a closed-form solution for JAFE.

**Figure 4:**
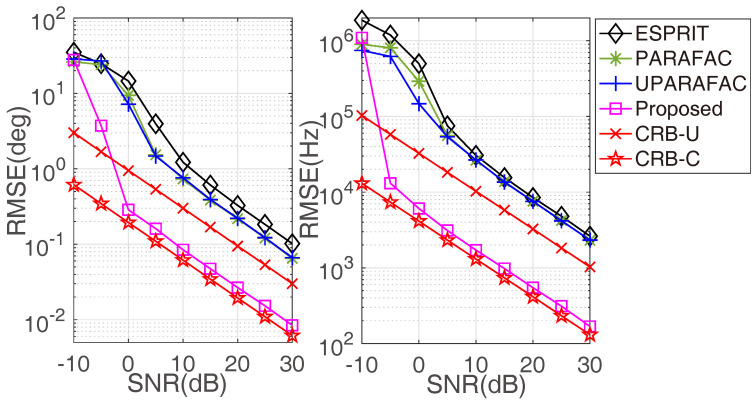
RMSE comparisons versus SNR.

Figure 4 depicts the RMSE of the proposed framework versus SNR, where $M_{1}=3$, $M_{2}=4$, $N_{1}=3$, $N_{2}=5$ and L=100. For comparison purposes, the RMSE curves with respect to ESPRIT [9], PARAFAC [10], UPARAFAC [11] and CRBs (CRBs that are associated with the ULA geometry and the coprime setup are marked with the suffixes ‘-U’ and ‘-C’, respectively) are added. Notably, the RMSE would be gradually improved with an increasing SNR. Additionally, the coprime configuration is inherent in providing a lower RMSE than the ULA geometry, as the CRB-C is much lower than CRB-U. As expected, the proposed framework outperforms all of the compared algorithms in both DOA and frequency estimation.

**Figure 5:**
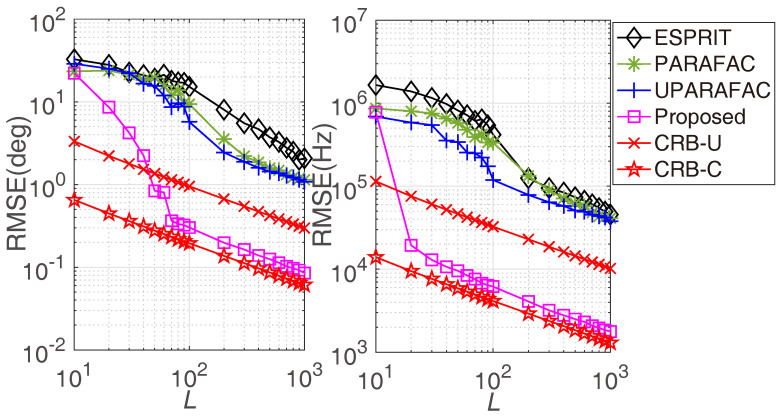
RMSE comparisons versus *L*.

Figure 5 illustrates the RMSE comparisons versus *L*, where $M_{1}=3$, $M_{2}=4$, $N_{1}=3$, $N_{2}=5$, and the SNR is fixed at 0 dB. Similar to the previous observation, a larger *L* would bring more accurate estimation results, since a larger *L* results in more accurate matrix/tensor decomposition. Additionally, it indicates that the proposed framework would achieve significantly lower RMSE than the traditional approaches when L≥20.

**Figure 6:**
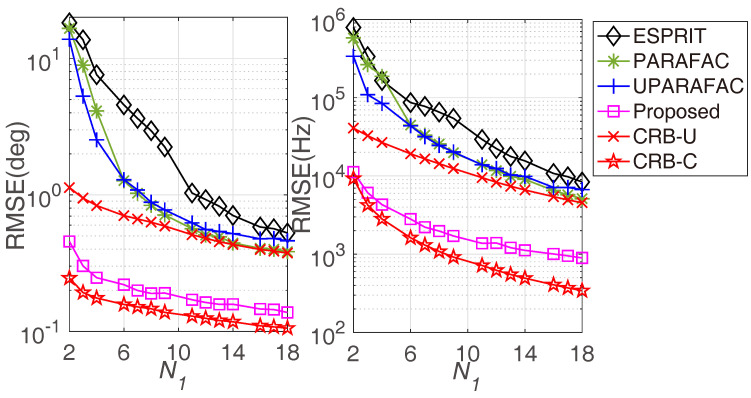
RMSE comparisons versus $N_{1}$.

**Figure 7:**
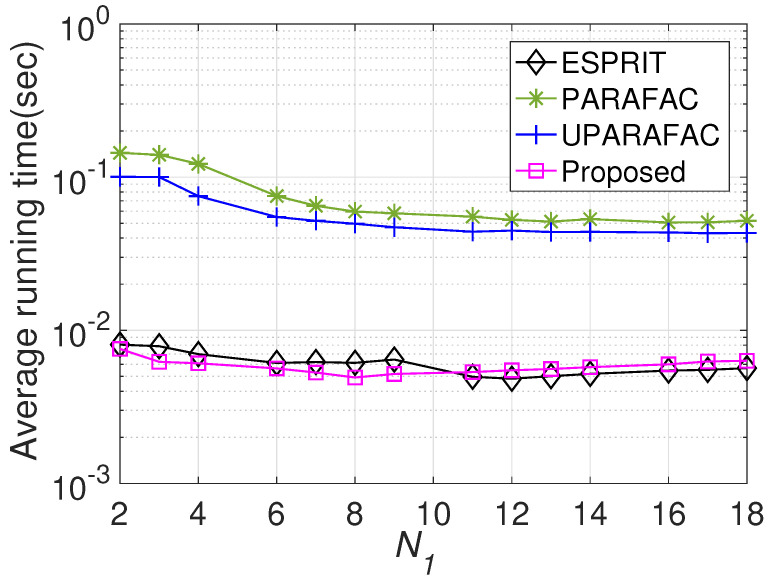
Average running time comparison versus $N_{1}$.

Figure 6 displays the RMSE comparisons with various $N_{1}$, where $M_{1}=3$, $M_{2}=4$, $N_{2}=5$, L=100, and the SNR is set to 0 dB. Additionally, a larger $N_{1}$ brings more accurate JAFE. Since the proposed framework occupies a larger temporal aperture, it obtains a remarkable improvement compared to the ULA-based approaches in the entire $N_{1}$ regions. In Figure 7, the average running time comparison with various $N_{1}$ is plotted. It seems that a larger $N_{1}$ has little impact on the average running time of various algorithms. As expected, the average running time of the proposed algorithm is very similar to that of the ESPRIT algorithm, but much shorter than the PARAFAC methods (both PARAFAC and UPARAFAC), as we stressed earlier.

**Figure 8:**
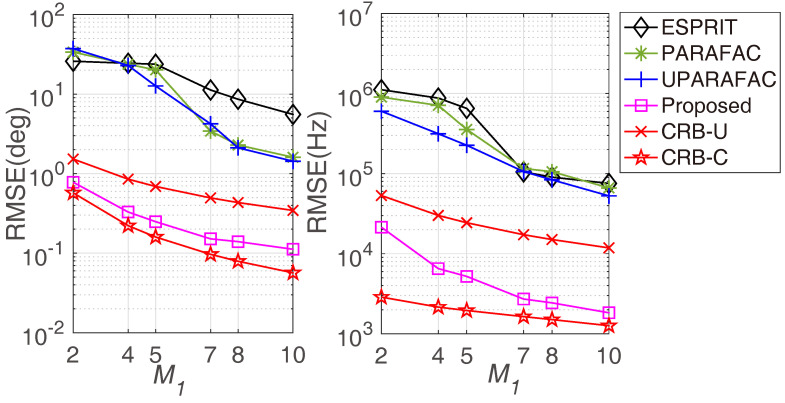
RMSE comparisons versus $M_{1}$.

**Figure 9:**
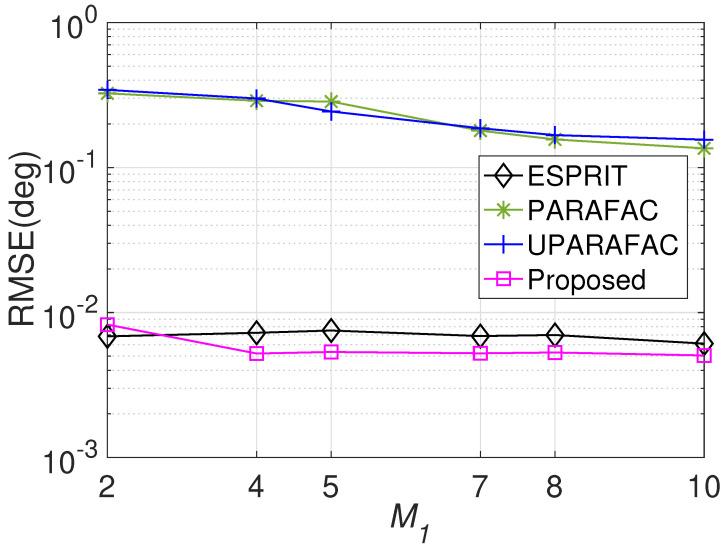
Average running time comparison versus $M_{1}$.

Figure 8 displays the RMSE comparisons with various $M_{1}$ values, where $M_{2}=3$, $N_{1}=3$, $N_{2}=5$, L=500, and the SNR is fixed at 0 dB. Similar to the previous observation, a larger $M_{1}$ brings more accurate results for JAFE, as a larger $M_{1}$ means a larger array spatial aperture as well as more degrees of freedom. Figure 9 reveals the average running time comparison with various $M_{1}$ values, which shows that the required running times of the proposed algorithm and the ESPRIT algorithm are much shorter than the tensor approaches. According to the simulations, it is suggested to adopt larger $M_{1}$, $M_{2}$, $N_{1}$ and $N_{2}$ in practice to obtain more accuracy estimation results.

## 6. Conclusions

In this paper, we have introduced a space–time coprime sampling framework for JAFE. A two-step ESPRIT algorithm has been developed for JAFE from the sub-Nyquist measurement. Benefiting from the larger spatial-temporal aperture of the coprime sampling, the proposed framework offers more accurate estimation performance than the current uniform sampling architectures. Simulation results agree with our theoretical findings. Since the proposed framework relies on a strict signal model and ignores the model errors, it may fail to work owing to imperfect scenarios, e.g., sensor position errors, non-ideal temporal sampling intervals or gain-phase errors. In the near future, more efforts should be devoted to JAFE with model errors, so as to enhance the robustness of the algorithm.

## Figures and Tables

**Table 1:** Algorithmic comparisons.

Algorithm	Spatial Aperture	Temporal Aperture	Complexity
ESPRIT	(M−1)d	(N−1)▵	$O\left\{M^{3}N^{3}\right\}+2{\left(M-1\right)}^{2}N^{2}K+\left(M-1\right)NK^{2}+2{\left(N-1\right)}^{2}M^{2}K+2{\left(N-1\right)}^{2}M^{2}K+\left(N-1\right)MK^{2}+O\left\{K^{3}\right\}$
PARAFAC	(M−1)d	(N−1)▵	$O\left\{M^{3}N^{3}\right\}+NLK^{2}+MLK^{2}+MNK^{2}$
UPARAFAC	(M−1)d	(N−1)▵	$O\left\{M^{3}N^{3}\right\}+0.25\left(NLK^{2}+MLK^{2}+MNK^{2}\right)$
Proposed	$\max\{M_{1}\left(M_{2}-1\right)d$, $M_{2}\left(M_{1}-1\right)d\}$	$\max\{N_{1}\left(N_{2}-1\right)▵$, $N_{2}\left(N_{1}-1\right)▵\}$	$O\left\{M^{3}N^{3}\right\}+2{\left(M_{2}-1\right)}^{2}N^{2}K+2{\left(M_{1}-1\right)}^{2}K+\left(M-1\right)NK^{2}+2{\left(N_{2}-1\right)}^{2}M^{2}K+2{\left(N_{1}-1\right)}^{2}M^{2}K+\left(N-1\right)MK^{2}+O\left\{K^{3}\right\}$

## Data Availability

Not applicable.
